# Vulnerability assessment to tropical cyclones in the North Caribbean Coast of Nicaragua (1988–2022)

**DOI:** 10.1371/journal.pone.0352206

**Published:** 2026-06-22

**Authors:** Lismaryin Muñoz-Requene, Pedro Sánchez-Zamora, Rosa Gallardo-Cobos

**Affiliations:** Department of Agricultural Economics, Higher Technical School of Agricultural and Forestry Engineering (ETSIAM), University of Cordoba, Rabanales University Campus, Córdoba, Spain; Universidade de Aveiro, PORTUGAL

## Abstract

The North Caribbean Coast Autonomous Region of Nicaragua (RACCN) is one of the most vulnerable areas to tropical cyclones (TCs), a condition exacerbated by climate change and internal structural disparities. Despite its historical exposure to TCs, significant gaps remain in the systematic analysis of its vulnerability. This research assesses the vulnerability levels of RACCN municipalities to TCs by constructing a Tropical Cyclone Vulnerability Index (VItc), and a municipal typology based on exposure, susceptibility, and adaptive capacity factors. Twelve indicators were selected, normalized, and analyzed using factor analysis with varimax rotation. The resulting sub-indices were integrated through weighted aggregation, and a municipal typology was developed using the Natural Breaks (Jenks) classification method. Findings reveal marked territorial disparities among municipalities. Bonanza exhibits high exposure and susceptibility combined with low adaptive capacity while Waslala demonstrates greater structural resilience. The VItc enabled the identification of three municipality clusters, with differentiated vulnerability profiles, facilitating the design of targeted interventions. The results underscore the role of social, demographic, and infrastructure factors in shaping local vulnerability. Despite limitations in data availability, the VItc provides a diagnostic tool that may help identify priority areas and the development of strategies for disaster risk reduction and resilience in the RACCN. The study highlights the need to improve data availability and strengthen institutional capacities. It also points to important directions for future research, including the integration of territorial, socioeconomic, and gender-sensitive approaches, as well as dynamic variables such as climate projections, land-use change, and migration, together with participatory methods that integrate local knowledge.

## 1. Introduction

Climate change represents one of the greatest global threats, with increasingly evident and widespread impacts [1]. It is associated with rising average ocean and atmospheric temperatures, sea level rise, and intensification of extreme weather events, including tropical cyclones (hereafter, TCs) [2], whose frequency and intensity have increased considerably over recent decades [3–5]. These phenomena disproportionately affect developing countries, particularly in the poorest local areas [6–8], generating food insecurity, water scarcity, forced displacement, economic losses, environmental degradation, infrastructure destruction, and species extinction [9]. According to the Index for Risk Management (INFORM), countries with higher disaster risks present high levels of poverty and limited capacity to respond to these events [10,11].

Vulnerability to TCs, understood as the degree to which a community or individual may be adversely affected by an extreme event due to the interaction between exposure, susceptibility, and the limited adaptive capacity to minimize its effects [12] depends not only on geographic exposure but also on socioeconomic, environmental, and institutional factors. Global research indicates that exposure to TCs, combined with characteristics such as population density, poor infrastructure, environmental degradation, and social inequality, significantly increases vulnerability in coastal areas [12,13]. In Latin America and the Caribbean, one of the regions most affected by natural disasters worldwide [14,15], vulnerability is shaped by structural conditions such as poverty, inequality, rapid population growth, political instability, and dependence on natural resources [16–18]. In Central America, these factors are combined with a high recurrence of extreme precipitation events, generating significant impacts on vulnerable communities, particularly in coastal areas [19–21].

Within this regional framework, Nicaragua faces structural conditions that increase its exposure to extreme events. With a Human Development Index of 0.669 [22] and high levels of poverty [23], the country is highly susceptible to floods, droughts, earthquakes, and particularly TCs [24]. The Autonomous Region of the Northern Caribbean Coast (hereafter, RACCN) has historically been one of the primary gateways for these events, resulting in significant human, material, and environmental losses [25].

Previous research in Nicaragua has addressed environmental [26], structural [27], and socioeconomic dimensions of vulnerability [24,28], while recent studies have also documented the historical occurrence of TCs [25,29]. Methodologically, macro-level vulnerability indices based on susceptibility, exposure, and adaptive capacity have been developed [30], alongside multiscale spatial analyses assessing hydrometeorological risk [31].

However, despite these advances, an integrated municipal-level assessment systematically analyzes the interaction between exposure, susceptibility, and adaptive capacity remains lacking, particularly in rural and coastal areas such as the RACCN. Addressing this gap, this research assesses the vulnerability levels of RACCN municipalities to TCs by constructing a composite vulnerability index and a municipal typology based on these three factors. This methodological approach aims to identify critical areas, generate updated empirical evidence, and provide key inputs for decision-making and the strengthening of regional resilience to climate change.

## 2. Conceptual framework

### 2.1 Evolution of the concept of vulnerability

Over time, the conceptualization of vulnerability has evolved significantly, both theoretically and in its practical application. Early approaches laid the foundation for risk analysis in human populations; however, they initially prioritized hazard characteristics over social conditions, minimizing the role of human factors in risk prevention and mitigation [32–35]. Subsequent research revealed that the political, economic, and social conditions of communities affected by natural hazards significantly influenced the magnitude and impact of disasters [32,34]. This shift led to a broader understanding of vulnerability as a condition shaped not only by external hazards but also by underlying structural inequalities.

Since the early 21^st^ century, the concept has gained greater recognition, mainly at the institutional level, where it has been defined as “the probability of being exposed to multiple risks, including natural disasters” and framed as one of the dimensions of human deprivation [36]. In the mid-2000s, theoretical debates intensified, and multiple assessment frameworks emerged, ranging from top-down approaches to bottom-up perspectives [37]. Today, vulnerability is a cross-cutting issue on the global agenda, linked to disaster risk reduction, sustainable development, climate resilience, and the fight against inequality.

#### 2.1.1 Conceptual approaches to vulnerability analysis.

The concept of vulnerability has been addressed from multiple disciplinary fields [33,34,38–43]. Consequently, its conceptualization varies according to the system under analysis, whether social [44], human-environmental [45,46], a specific population group, an economic sector, a geographic region, or a defined temporal period, as well as the type of disturbance or stress factor considered [43].

Three predominant approaches to conceptualizing and assessing vulnerability have been identified. The first, known as the *natural hazards* approach [47,48], addresses vulnerability from a perspective focused on risk perception, and natural hazards science, conceptualizing it as the potential for loss or experiencing adverse effects, i.e., the capacity to suffer harm [49].

The second approach corresponds to *social constructivism* with roots in human geography [50], political ecology [51,52], political economy, and sociology [53]. It considers vulnerability as a pre-existing structural condition determined by socioeconomic, historical, and political factors [51,54,55]. In contrast to the natural hazard-centered perspective, vulnerability involves a combination of non-climatic structural factors and pre-existing conditions that increase the susceptibility of certain communities to the adverse impacts of disasters [34].

The third approach, characterized by its integrative perspective, emerges from the framework of *social-ecological systems*, defined as complex, integrative, and adaptive systems [56], composed of the interactions between humans and the environment, with characteristics of complexity [57,58]. In this approach, vulnerability is understood as an emergent property of the system, structured from three key components: exposure, referring to the degree to which a social system is exposed to external threats; susceptibility, related to the internal conditions of the system; and resilience, the system’s capacity to cope with, respond to, and adapt to the effects of a disruptive event [59–61].

#### 2.1.2 Vulnerability in the context of climate change.

Vulnerability research applied to climate change is highly interdisciplinary, involving both natural and social sciences in its measurement and assessment [62]. In the reports of the Intergovernmental Panel on Climate Change (IPCC), the conceptualization of vulnerability has evolved over time, reflecting advances in research and the perspectives adopted by the authors who have addressed it [63]. According to Birkmann, there is no universally accepted definition of vulnerability in this context [64], and it is considered a function of several biophysical and socioeconomic factors [65]. Along the same lines, Ford and Smit define it as a function or reflection of the exposure of affected communities to climate change and their adaptive capacity, i.e., their ability to adjust to and manage its effects [66].

Vulnerability is now a concept institutionalized by both the IPCC and the United Nations Development Programme (UNDP). In its Fifth Assessment Report, the IPCC adopts an approach based on social-ecological systems, defining vulnerability as “the propensity or predisposition [of a system] to be negatively affected,” encompassing elements such as susceptibility to harm and limited capacity to adapt [18,49]. According to this conception, vulnerability has three main components: exposure, susceptibility, and adaptive capacity.

In this conceptual framework, *exposure* refers to the presence of people, environmental functions, services, resources, infrastructure, or assets in places that could be adversely affected by climatic hazards. *Susceptibility* refers to the internal characteristics of the system that increase its sensitivity to the hazard in question. *Adaptive capacity* is defined as the ability of the system to resist, recover, and adjust to that stress factor [41,46,67]. Throughout this study, the terminology follows the conceptual framework adopted by the IPCC, using the components exposure, susceptibility, and adaptive capacity.

Therefore, vulnerability can be understood as the result of the interaction between potential impacts, which combine the key components of exposure and susceptibility, and the system’s capacity to adapt. Thus, various studies have represented this relationship using a widely used conceptual formulation: *Vulnerability (V) = f (exposure (E), susceptibility (S), adaptive capacity (CA))* [40,68–70], whose calculation, in operational terms, can be expressed through the following equation: V=E+S−CA

### 2.2 On the assessment of vulnerability

The diversity of disciplines addressing the concept of vulnerability has led to the development of multiple methodological frameworks for its assessment. The selection of the methodology depends on how vulnerability is conceptualized, as well as on the purpose of the study and the scales of assessment (spatial, temporal, and decision-making) [43,71]. In the 21st century, an approach based on mixed methods, which combines qualitative and quantitative techniques, has predominated [34,72,73]. In this framework, the incorporation of approaches that integrate quantitative indicators and spatial analysis of the context becomes particularly relevant in dispersed rural territories such as the RACCN. These integrative approaches aim to communicate the concept of the “vulnerability of place,” which refers to the potential for harm arising from the interaction between climatic events and the social and territorial conditions of the local context [40,74].

One of the most widely used methodologies for measuring vulnerability is the construction of composite indices, which enable the relative quantification of vulnerability within a given territory. According to De Sherbinin et al. [75] in a systematic review of studies on vulnerability to climate change and TCs, the construction of indices is the most widely used method for data aggregation. These indices are useful tools, both conceptually and operationally, as they provide a relative quantification of the state of vulnerability [76].

However, the construction of vulnerability indices involves a series of methodological decisions that can influence the results obtained. These include the selection of variables, data availability, the scale of analysis, and the methods of normalization, aggregation, and weighting of indicators [77]. In this regard, authors such as Holand and Lujala recommend adapting the methodological approach to the specific circumstances of the study area rather than adhering to rigid formulas [78], since the selection of variables is, to a certain extent, a subjective process, and different combinations of indicators can generate significantly different vulnerability patterns [79]. Therefore, variables and datasets must be adjusted to the availability and specific needs of the study area [80].

Regarding the selection of indicators, considering that vulnerability is determined by exposure to climate change, susceptibility, and adaptive capacity of a system, the literature offers variables commonly associated with each sub-index. For exposure, indicators that reflect the presence of populations or assets in potentially affected areas, the intensity and physical presence of the climate phenomenon are often used [68,81,82].

To measure susceptibility, studies agree on the use of social and demographic variables that increase vulnerability to disasters [42,74,78,79,81,83,84]. Concerning to adaptive capacity, the literature emphasizes variables related to the availability of institutional, physical, and community infrastructure to face extreme events and adjust to damage [81,85–87].

Finally, vulnerability studies often present maps of individual indicators, and to a lesser extent, of aggregated components. Spatial mapping contributes to identifying critical areas for developing effective disaster risk reduction and adaptation strategies [75]. This tool is essential for guiding mitigation actions and territorial planning, as well as for enhancing communication with policymakers and local actors [71]. In this context, the present study adopts a composite index approach to assess vulnerability to tropical cyclones in the municipalities of the RACCN, structured around the three components proposed by the IPCC: exposure, susceptibility, and adaptive capacity

## 3. Materials and methods

### 3.1 Study area

The geographical scope of this research is the North Caribbean Coast Autonomous Region of Nicaragua (RACCN), one of the largest administrative units in Nicaragua. The analysis focuses on the recurrence of TCs in this region over a 32-year period (1988–2022) to determine their level of vulnerability. The territorial unit of analysis is the municipality.

**Fig 1:**
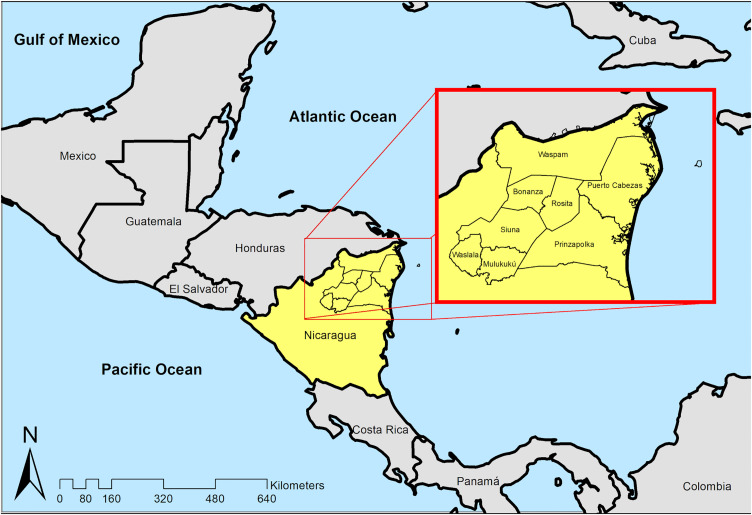
Map of the study area. **(a)** North Caribbean Coast Autonomous Region of Nicaragua (RACCN); **(b)** Municipalities within the RACCN.

The RACCN has a surface area of 33,105.98 km², and according to municipal censuses data reported to Nicaragua’s National System for Disaster Prevention, Mitigation and Attention (SINAPRED, by its Spanish acronym), its estimated population for 2024 is 712,601 inhabitants. It is composed of eight municipalities: Bonanza, Siuna, and Rosita (known as the mining triangle), Mulukukú, Puerto Cabezas, Prinzapolka, Waslala, and Waspám (Fig 1).

According to the Nicaraguan Institute of Territorial Studies (INETER), the RACCN presents a humid climate, with an average annual rainfall of 2,500 mm and temperatures ranging between 26.0°C and 28°C. The population is mostly young and rural, with a distribution of 65% in rural areas and 35% in urban areas. It also stands out for its ethnic diversity, with Miskito, Mestizo, Mayangna, and Creole peoples. Table 1 summarizes key territorial and demographic data by municipality, including surface area, total population, urban-rural distribution, and population density.

**Table 1:** Data by municipalities of the RACCN.

Municipality	Area	Total population	% Urban Population	% Rural Population	Population density
**Bonanza**	1,897.94	85,654.00	46	54	45
**Mulukukú**	1,904.53	60,793.00	20	80	32
**Prinzapolka**	7,020.48	48,275.00	9	91	7
**Puerto Cabezas**	5,984.81	158,621.00	57	43	27
**Rosita**	2,205.42	101,713.00	32	68	46
**Siuna**	3,421.58	100,820.00	21	79	29
**Waslala**	1,329.51	52,813.00	51	49	40
**Waspám**	9,341.71	103,912.00	19	81	11
**Total, RACCN**	33,105.98	712,601.00	35	65	22

Source: SINAPRED- Data updated to 2024.

Economic activities in the RACCN vary according to the geographic location of the municipalities, although the region is generally characterized by a high dependence on the primary sector. Puerto Cabezas and Prinzapolka are primarily dedicated to fishing. At the same time, Bonanza, Siuna, and Rosita combine mining, agriculture, and cattle ranching, with the latter two activities being pursued in lesser proportions. In Mulukukú, cattle ranching is predominant, followed by the cultivation of staple grains such as corn and beans. In Waslala, the local economy has traditionally been based on agriculture, although cattle ranching has gained importance in recent years. Waspám’s economy is based on subsistence agriculture, livestock, and timber exploitation, with small segments of the population dedicated to artisanal gold panning, known in the region as “güirisería”, and artisanal fishing in rivers and lagoons.

In terms of road infrastructure, Nicaragua’s national road network totaled 24,781 km in 2023, of which approximately 8% corresponded to the RACCN. This network includes paved, asphalted, hydraulic concrete, and cobblestone sections. The road infrastructure is composed of secondary trunk roads (305.23 km), which connect departmental capitals and relevant economic centers, facilitating interdepartmental and regional transport flows; primary collector roads (117.37 km), which connect one or more municipalities and population centers not directly served by the trunk network; secondary collector roads (177.66 km), which link intermediate population centers and minor traffic generators with the main collector or trunk routes, playing an important role at the municipal level; and rural access roads (1,399.37 km), which represent approximately 70% of the total and provide local access to rural communities and remote areas, facilitating the connection of productive areas and dispersed settlements with higher-level roads and with consumption and export centers [88].

The municipalities of the RACCN face high levels of poverty, marginalization, and social exclusion [89]. Due to the region’s high exposure to extreme weather events, the region has repeatedly been designated a priority area for intervention by international organizations, including various United Nations offices, the Central American Bank for Economic Integration (CABEI), the World Food Programme (WFP), the International Federation of Red Cross and Red Crescent Societies (IFRC), development cooperation agencies, and non-governmental organizations (NGOs). This presence reflects the magnitude of the humanitarian impacts that tropical cyclones have historically generated in the territory, especially during the emergency phase.

#### 3.1.1 Tropical cyclones in the RACCN.

The National Oceanic and Atmospheric Administration (NOAA) defines a “tropical cyclone” as a low-pressure system that forms over tropical waters (between 25°S and 25°N), with storm activity near the center of its closed and cyclonic winds. Its classification depends on the intensity of maximum sustained winds: tropical wave or disturbance (closed circulation), tropical depression (≤ 38 mph), tropical storm (39–73 mph), and hurricane (≥74 mph). Hurricanes are further categorized according to the Saffir-Simpson scale in five levels based on wind intensity: category 1 (74–95 mph), category 2 (96–110 mph), category 3 (111–129 mph), category 4 (130–156 mph), and category 5 (≥157 mph).

According to the review of the Emergency Events Database (EMDAT) and reports from NOAA’s Hurricane Research Division, during the period between 1988 and 2022, Nicaragua recorded a total of 30 cyclonic events. The table in S1 Table lists the TCs recorded in Nicaragua during the study period, including their name, category, month, and year of occurrence, as well as the region of impact (landfall). The months with the highest recurrence were October (10 events), September (6), November (5), May (3), and August, June, and July (2 each). Of the 30 events identified, 3 were classified as tropical disturbances or waves (WL), 5 as tropical depressions (TD), 12 as tropical storms (TS), and 10 as hurricanes (H). Of the latter, 3 were category 1, 2 were category 3, 3 were category 4, and 2 reached category 5, considered super hurricanes due to their intensity.

Of the total, 11 made landfall in the RACCN, 8 in the Autonomous Region of the Southern Caribbean Coast (RACCS), 2 in the Pacific region, and 1 through Honduras. In addition, 8 events, although they did not make direct landfall in the RACCN, caused significant impacts in this region due to their close trajectory over the Caribbean Sea.

### 3.2 Phases, sources, and tools of analysis

**Fig 2:**
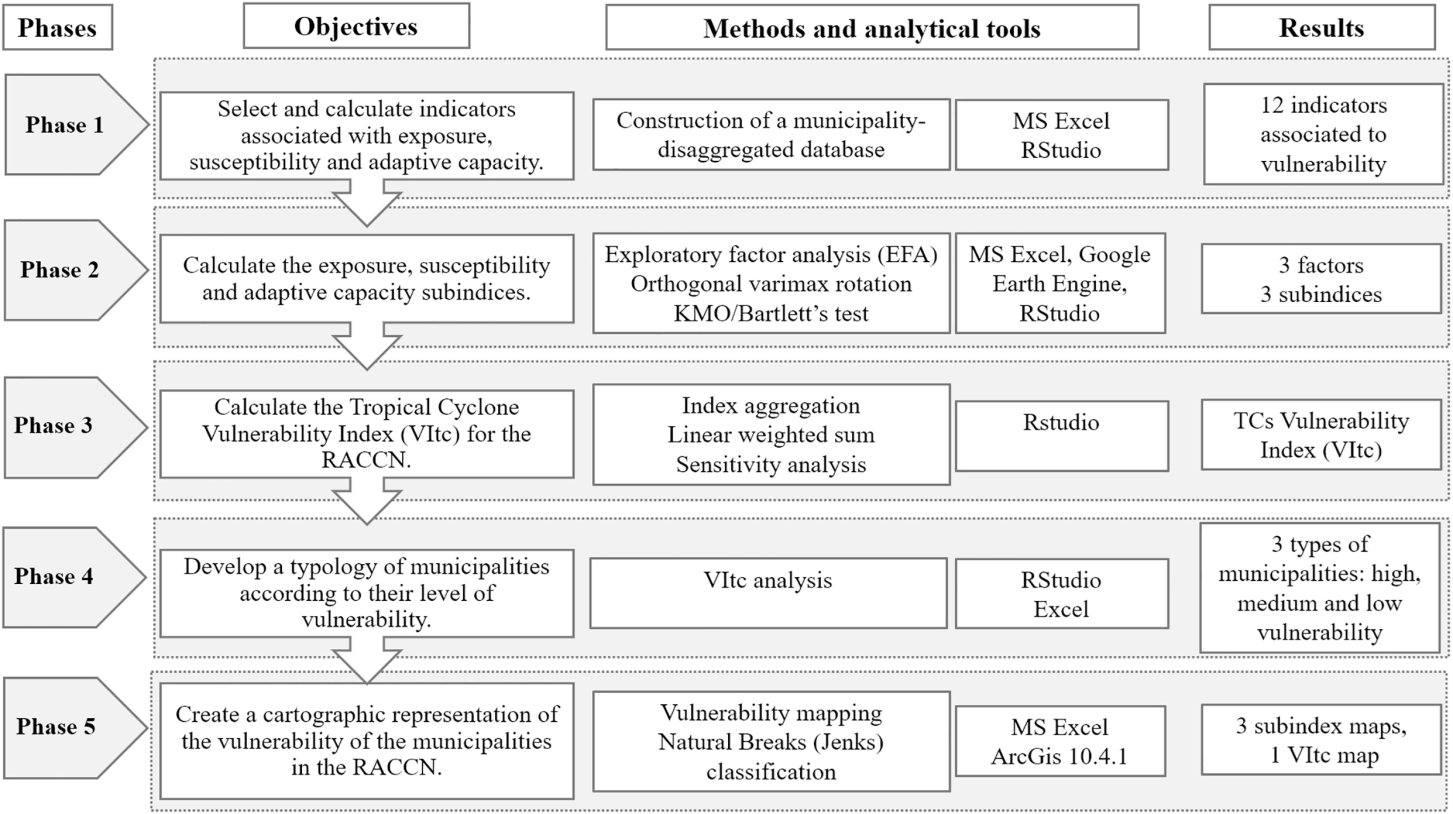
Methodological framework. Diagram illustrating the five research phases, including the methodological tools applied and the expected results for each phase.

This study was developed in five fundamental phases. The methods and tools used, as well as the expected results for each phase, are shown in (Fig 2).

#### 3.2.1 Phase 1: Selection of vulnerability indicators.

The first phase involved the selection of indicators used to construct the sub-indices of the Vulnerability Index to Tropical Cyclones (VItc). This selection was guided by the adopted theoretical framework and the availability of reliable municipal-level statistical data from official sources. However, local stakeholders and community representatives were not directly involved in defining the variables included in the index, which is acknowledged as a limitation of this study.

In total, 12 indicators were selected, distributed across the three components of vulnerability: exposure, susceptibility, and adaptive capacity, as shown in S2 Table. Each indicator was defined based on its relevance to disaster risk assessment and its ability to reflect structural and contextual vulnerabilities. The exposure component includes three indicators associated with cyclonic events and the presence of a potentially exposed population in the territory. These indicators are: cumulative precipitation by municipality (EXP1PA) derived from cyclonic events (unit: mm/km², source: EM-DAT and National Hurricane Center); density of critical points (EXP2PC) (unit: PC/km²; source: SINAPRED); and population density (EXP3DP) expressed as the number of inhabitants per square kilometer (unit: people/km²; source: SINAPRED. In terms of directionality within the index, higher values of these indicators are interpreted as an increase in the level of exposure and, therefore, in the level of vulnerability.

The susceptibility component incorporates five sociodemographic indicators that reflect the presence of population groups that are potentially more vulnerable to the impacts of tropical cyclones. These indicators are: rural population (SC1PR) expressed as a percentage of the total municipal population (unit: %; source: SINAPRED); pregnant women (SC2PG) (unit: %; source: SINAPRED); children under five years of age (SC3CH) (unit: %; source: SINAPRED); adults over 65 years of age (SC4EL) (unit: %; source: SINAPRED); and people with disabilities (SC5DIS) (unit: %; source: SINAPRED). In terms of directionality within the index, higher values of these indicators are interpreted as an increase in the level of susceptibility and, therefore, in the level of vulnerability.

**Fig 3:**
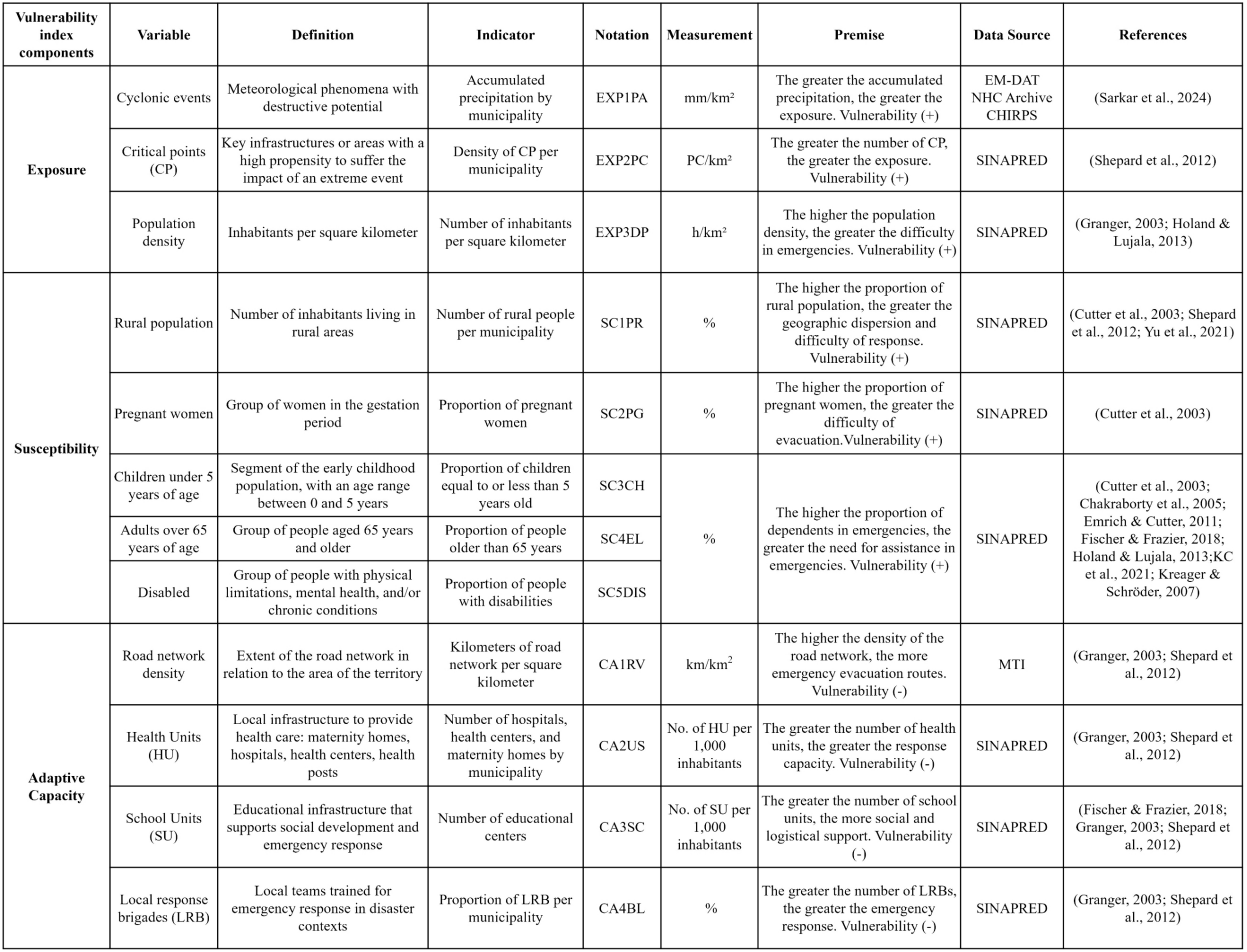
Vulnerability indicators by component. Overview of the indicators selected for exposure, susceptibility, and adaptive capacity sub-indices, including their definitions, measurement units, data sources, and references.

Finally, the adaptive capacity component includes four indicators related to the availability of basic infrastructure and institutional and community capacities for preparedness and response to extreme events. These indicators are: road network density (CA1RV), measured as kilometers of road per square kilometer (unit: km/km²; source: MTI); health facilities (CA2US) (unit: number of facilities per thousand inhabitants; source: SINAPRED); educational institutions (CA3SC) (unit: number of educational institutions per thousand inhabitants; source: SINAPRED); and local brigades (CA4BL) (unit: %; source: SINAPRED). Higher values of these indicators are interpreted as higher levels of adaptive capacity and, therefore, as factors that contribute to reducing the level of vulnerability. A synthetic overview of the indicators used in the construction of the vulnerability sub-indices is presented in (Fig 3).

The statistical information used in this study was obtained from the following sources:

*The Emergency Events Database (EMDAT)*, administered by the Center for Research on the Epidemiology of Disasters (CRED), provides an international record of disasters from 1900 to the present [90,91].

*The National Hurricane Center (NHC) Archive,* which contains detailed reports on each of the TCs, including name, start and end dates, historical synopsis, track, intensity, and associated meteorological data.

*Climate Hazards Group InfraRed Precipitation with Station data (CHIRPS)*, a database of accumulated precipitation derived from cyclonic events. It combines satellite imagery and in situ station data to create gridded precipitation time series. Almost all data have a spatial resolution of 0.05° × 0.05° [92–96].

Additionally, *RACCN’s sociodemographic* data on vulnerable populations (pregnant women, people with disabilities, children under five, and adults over 65), as well as data on social infrastructure (local brigades, health units, and educational centers), have been provided by SINAPRED. This entity articulates information from different ministries and public sector institutions in Nicaragua. Data on road network density was obtained from public sources of the Ministry of Transportation and Infrastructure (MTI). All this information was integrated into a disaggregated database at the municipal level, composed of a total of 12 indicators.

#### 3.2.2 Phase 2: Calculation of vulnerability sub-indices.

The calculation of the values corresponding to the exposure, susceptibility, and adaptive capacity sub-indices was conducted following the methodological guidelines proposed by Nardo et al [97]. Once the variables and indicators were selected, the data were normalized using the min-max method, adjusting their values to a range from 0 to 1. This technique ensures comparability between indicators measured on different scales, facilitating their integration into the composite index [98].

In this study, the assignment of indicators to the components of exposure, susceptibility, and adaptive capacity followed an inductive–deductive approach. In the first step, the indicators were theoretically assigned to each component based on the adopted conceptual framework and the relevant literature on vulnerability. Subsequently, this classification was compared with the empirical structure derived from exploratory factor analysis (EFA). When the empirical grouping matched the theoretical classification, the indicators were retained in their original component. When discrepancies arose, the final assignment was determined by considering the statistical association of the indicator with the corresponding factor.

Prior to factor extraction, the suitability of the dataset for factor analysis was evaluated using the Kaiser–Meyer–Olkin (KMO) measure and Bartlett’s test of sphericity, which assess whether the correlations among variables are adequate for factor analysis [99]. Likewise, the communalities of the indicators were evaluated to determine the proportion of variance explained by the common factors. High communalities indicate that a substantial proportion of the variance of the observed variables is explained by the common factors [100,101].

Subsequently, exploratory factor analysis was conducted to identify the latent structure among the indicators and estimate the weights used in the construction of the sub-indices. Factor extraction was performed using the maximum likelihood method. To facilitate the interpretation of the retained factors, varimax orthogonal rotation was employed. Once the factor loadings were identified, the indicators within each sub-index were weighted accordingly*.* The weights were calculated by multiplying the normalized values of each indicator by their respective factor loadings, understood as their statistical contribution to the latent factor. Finally, each sub-index was calculated by summing the weighted products and subsequently rescaled to ensure comparability between municipalities. This operation was performed according to the following formula:


$$\begin{array}{c} {Ind}_{Pj}=\sum \overset{n}{\underset{i=\text{1}}{\mathrm{\backslash nolimits}}}\left(\overset{norm}{\underset{i}{v}}\times C_{i}\right) \end{array}$$

Where:

${Ind}_{Pj}$, is the sub-index value for component *j,* which can be Exposure *(E),* Susceptibility *(S),* and Adaptive Capacity *(AC).*

${v_{i}}^{norm}$ is the normalized value (min-max) of indicator *i*, for each observation.

*C*_*i*_ is the factor loading of indicator *i*, i.e., its contribution to the latent factor.

*n* is the total number of indicators considered for each component.

#### 3.2.3 Phase 3: Calculation of the Tropical Cyclone Vulnerability Index (VItc).

The Tropical Cyclone Vulnerability Index (VItc) for the eight municipalities of the RACCN was calculated from the weighted combination of the sub-indices exposure *E*, susceptibility *S*, and adaptive capacity *CA* sub-indices. The exposure and susceptibility sub-indices were summed together, as an increase in any of them contributes to greater overall vulnerability. In contrast, adaptive capacity (AC) was subtracted since a greater adaptive capacity reduces the impact of the TCs. The formula for its calculation is as follows:


$$\begin{array}{c} VItc=W\text{1}\times E+W\text{2}\times S-W\text{3}\times CA \end{array}$$

Where:

*VItc* represents the TC Vulnerability Index.

*E, S, and CA*: sub-indices of exposure, susceptibility, and adaptive capacity, respectively.

*W1, W2, W3* correspond to the weights assigned to each sub-index, calculated as the proportion of variance explained by each factor, according to factor analysis [79,102].


$$\begin{array}{c} Wn=\frac{\%\;explained\;variance}{total\;variance} \end{array}$$

The VItc provides a synthetic measure of the level of vulnerability of the municipalities on a scale of 0–1, where 0 is the lowest vulnerability and 1 the highest, within the set of evaluated municipalities. In order to assess the robustness of the constructed index, a sensitivity analysis was performed using principal component analysis (PCA) with jackknife and bootstrap resampling procedures. This procedure allows the stability of the index weights to be examined in the face of variations in the data and verifies the consistency of the vulnerability patterns obtained using different estimation methods.

#### 3.2.4 Phase 4: Typology of municipalities according to level of vulnerability.

A typology of municipalities was established based on the values obtained for the Tropical Cyclone Vulnerability Index (VItc), to identify groups of municipalities with differentiated patterns of vulnerability. This classification facilitates comparative analysis and the formulation of differentiated intervention strategies in scenarios involving extreme events and the management of risks associated with TCs.

#### 3.2.5 Phase 5: Mapping the level of vulnerability of the RACCN’s Municipalities.

Finally, the results obtained for the sub-indices, the VItc, and the municipal typology were analyzed and interpreted. For cartographic representation, spatial layers were generated in ArcGIS 10.4.1 software, corresponding to the sub-indices, the VItc, and the typology of municipalities. The index values were grouped into three classes (low, medium, and high), using the Natural Breaks (Jenks) classification method. This method allows the identification of natural groupings in the data by minimizing the intra-class variance and maximizing the variance between classes. Since the data presents a clustered distribution around certain values (the result of the underlying factors analyzed), this technique is more appropriate than other conventional classification methods, such as quantiles, standard deviation, or equal intervals, as it reveals patterns that are not obvious through simple observation [102].

However, to assess the sensitivity of the classification to the limited number of territorial units analyzed (n = 8), a methodological comparison was also conducted using three cartographic classification schemes: Natural Breaks (Jenks), Quantiles, and Standard Deviation. This comparison made it possible to evaluate the stability of the class break values and the resulting spatial pattern of the vulnerability index.

## 4. Results and discussion

This section presents the results of the factor analysis and the VItc calculated from the exposure, susceptibility, and adaptive capacity sub-indices. It also establishes the typology of the RACCN municipalities according to their levels of vulnerability.

### 4.1 Factor analysis results: Factor extraction and indicators grouping

Exploratory factor Analysis identified three factors that together explain 93.8% of the total variance of the dataset. Previously, the suitability of the data for factor analysis was evaluated using the Kaiser–Meyer–Olkin (KMO) measure and Bartlett’s sphericity test. The overall KMO value was 0.605, indicating moderate suitability for factor analysis. Likewise, Bartlett’s sphericity test was statistically significant (χ² = 48.133; p < 0.001), confirming that the correlation matrix presents sufficient associations between the variables to proceed with factor extraction.

**Fig 4:**
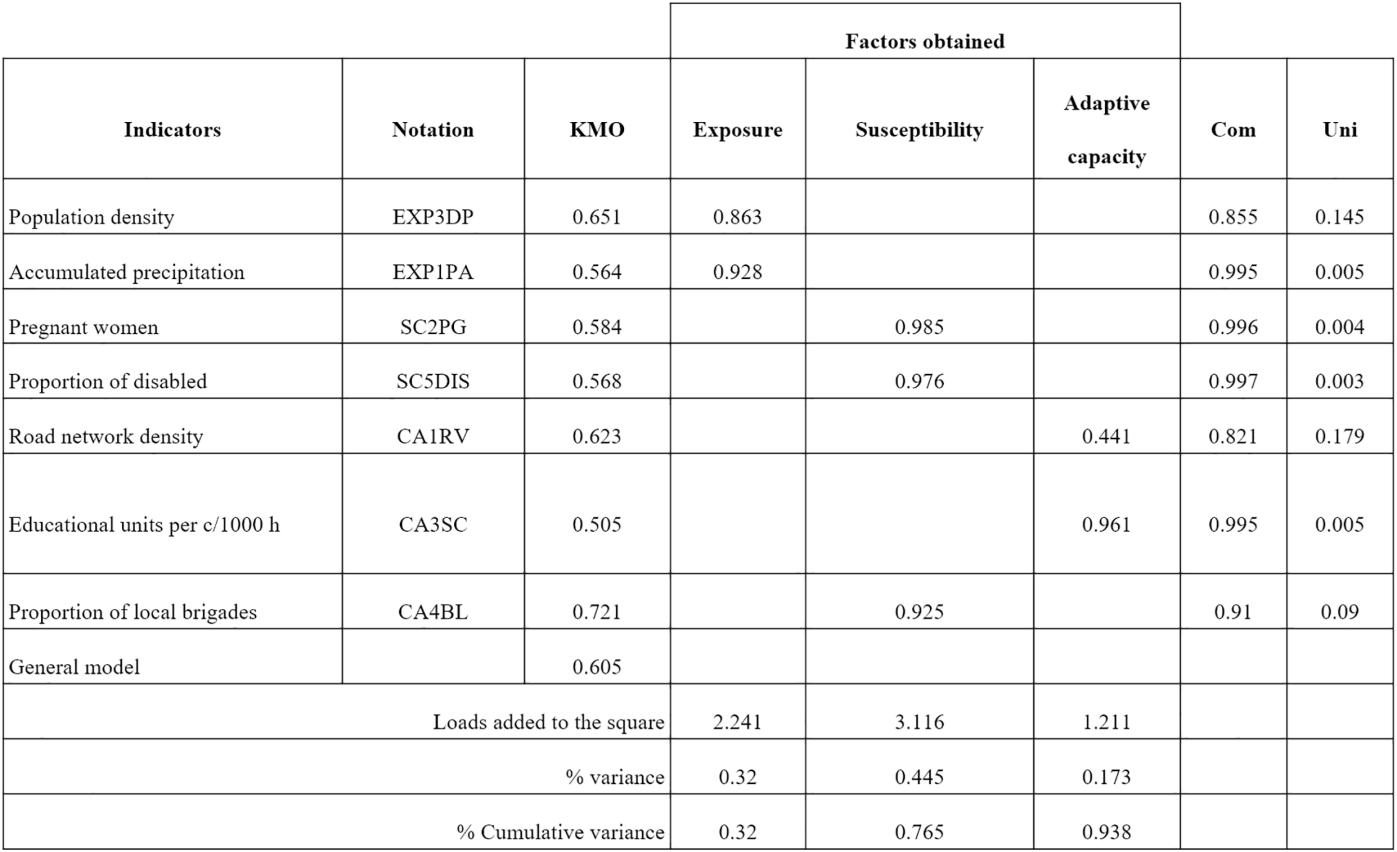
Matrix of components and factor loadings. Results of the exploratory factor analysis, showing the factor loadings of each indicator with respect to the three components of vulnerability: exposure, susceptibility, and adaptive capacity. The matrix also includes the Kaiser-Meyer-Olkin (KMO) values, communalities (Com), and uniqueness (Uni) for each variable. Only factor loadings higher than 0.4 are reported.

Additionally, the communalities of the indicators were high, ranging from 0.821 to 0.997, indicating that a substantial proportion of the variance is explained by the common factors. This result supports the stability of the factorial solution. The factor loadings for each indicator, along with their respective KMO, communality (Com), and uniqueness (Uni) values, allow for the validation of their contribution to the model, and the quality of the fit are presented in (Fig 4).

The first factor, named susceptibility (44.5% of the explained variance), includes the indicators of proportion of pregnant women (0.985), proportion of people with disabilities (0.976), and proportion of local brigades (0.925). The indicator related to the presence of local brigades was initially conceptualized as a component of adaptive capacity, reflecting the organizational capacity of communities to respond to disaster events. However, in the EFA, this variable exhibited a stronger statistical association with the susceptibility component. Following the inductive–deductive decision rule adopted in this study, the final assignment of the indicator was based on its empirical loading structure while acknowledging its conceptual link to community preparednes. For this reason, the indicator was retained within the susceptibility factor, inverting its scale through the transformation (1 - CA4BL). This transformation ensures internal consistency within the factor, where all indicators contribute positively to increased vulnerability.

The second factor, named exposure (32% of the variance explained), groups the indicators of accumulated precipitation (factor loading = 0.928), population density (0.863), and road network density (0.729). Although population density was theoretically assigned to the susceptibility component, its strong statistical association with the exposure factor is methodologically valid, given that a higher population concentration implies greater physical exposure to TCs. This reassignment finds support in studies which evidence how certain indicators traditionally linked to susceptibility may functionally relate to exposure, by reflecting the interaction between the social system and the environmental system [87]. In other words, this responds to the relationship between humans and their environment, recognizing the interrelated and dynamic nature of components in complex social-ecological systems [46,57,58].

The third factor extracted, named to adaptive capacity, explains 17.3% of the variance. This factor groups the indicators of the number of educational units per thousand inhabitants (0.961) and the density of the road network (0.441), both of which are consistently assigned to the component from both a theoretical and statistical perspective. These indicators represent structural and functional elements that contribute to reducing vulnerability to TCs by facilitating access to essential services and mobility in emergency contexts.

The adjustments made between the initial theoretical assignment of the indicators (deductive approach) and their empirical grouping after the factor analysis (inductive approach) do not undermine the conceptual classification proposed for each vulnerability dimension. On the contrary, they enhance the construction of the composite index by integrating emerging data patterns with the underlying theoretical framework. This mixed-method integration aligns with the complexity inherent in the development of composite indicators. As noted by Nelson et al. [103] and Pelling [104], deductive and inductive approaches should not be understood as mutually exclusive, but rather as complementary, as they tend to converge in the applied stages of the process (as in the construction of the VItc in this study). The results obtained support this methodological vision, highlighting its relevance for accurately representing vulnerability in specific territorial contexts.

The results of factor analysis also identified susceptibility as the factor with the greatest explanatory weight in the construction of the vulnerability index. These findings are consistent with other previous studies in the field [84,102], which has assigned a predominant weight to the dimension associated with the vulnerable population, referred to as *susceptibility* or *sensitivity*, depending on the terminology used by each author.

### 4.2 Vulnerability assessment and analysis

#### 4.2.1 Exposure sub-index.

**Fig 5:**
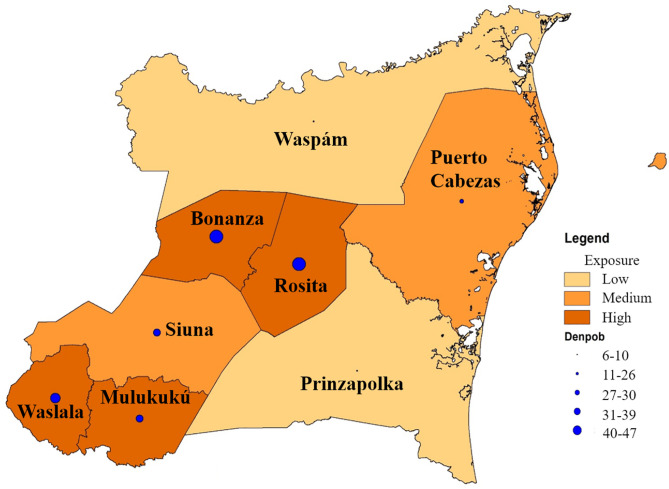
Map of exposure sub-index. Spatial distribution of the exposure sub-index across the eight municipalities of the North Caribbean Coast Autonomous Region of Nicaragua (RACCN). Darker areas of the map reflect a higher degree of exposure to tropical cyclones based on accumulated precipitation and population density.

The exposure factor, composed of the indicators of accumulated precipitation and population density, represents the degree of contact of the population with the TCs. Details on the methodology used to calculate the accumulated precipitation indicator are provided in S1 Appendix. The results indicate that the municipalities of Bonanza, Mulukukú, Rosita, and Waslala present the highest exposure values (close to or above 0.70). Siuna and Puerto Cabezas are located at a medium level, while Waspám and Prinzapolka register the lowest levels. This spatial pattern is illustrated in (Fig 5).

The observed patterns are consistent with historical records of accumulated precipitation in the region (see S2 Appendix) and with the spatial distribution of population density. During the 32 years analyzed, the municipalities of Rosita, Bonanza, Waslala, and Mulukukú registered significant volumes of accumulated precipitation, which, combined with their high population density, increases their level of exposure.

Incorporating population density into cartographic visualization enables the identification of the potential number of people exposed to TCs. This overlap reveals that municipalities with high precipitation but low population density, such as Siuna and Puerto Cabezas, have intermediate levels of exposure. On the other hand, although Waspám and Prinzapolka are among the municipalities with the highest accumulated volumes of precipitation during the analyzed cyclonic events, their level of exposure is low. This apparent contradiction is explained by their low population density, which significantly reduces the number of people potentially affected by the direct impact of these phenomena.

According to the theoretical framework adopted, exposure depends not only on the intensity of the TCs but also on the presence and concentration of the population in the affected areas. This study reaffirms that exposure is a relational component, conditioned by both the characteristics of the hazard and the degree of occupation and use of the territory. In this study, population density is used as a proxy for demographic exposure at the municipal level, reflecting the potential concentration of people that may be affected by cyclone impacts rather than a direct measure of physical exposure to the Hazard. Previous studies have demonstrated that higher population density significantly increases the degree of exposure to extreme precipitation events [105–107]. Similarly, densely populated areas have been found to exhibit higher vulnerability to TCs compared to less populated regions [108]. Therefore, the occurrence of high precipitation in territories with low population concentration, as is the case of Waspám and Prinzapolka, does not necessarily imply high demographic exposure to risk, as evidenced by the results of this analysis.

#### 4.2.2 Susceptibility sub-index.

**Fig 6:**
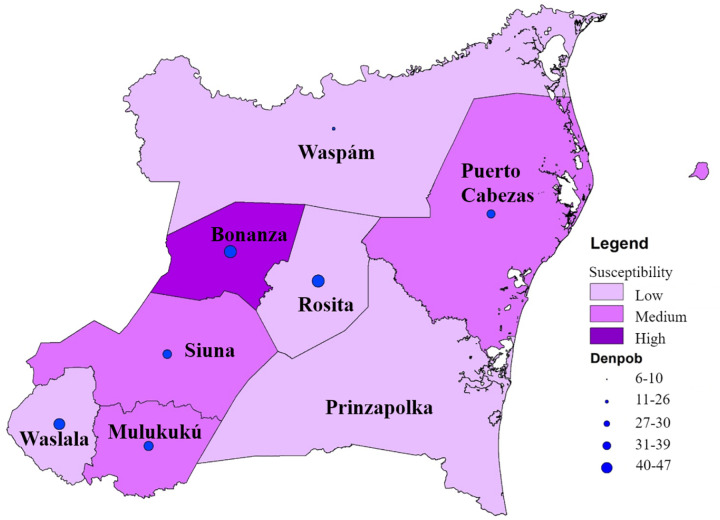
Map of susceptibility sub-index. Spatial representation of the susceptibility sub-index for the eight municipalities of the North Caribbean Coast Autonomous Region of Nicaragua (RACCN). Darker areas denote higher susceptibility to the impacts of tropical cyclones.

The susceptibility sub-index is composed of three indicators: the proportion of pregnant women, the proportion of people with disabilities, and the local brigades. Except for the latter, the first two describe inherent characteristics of the vulnerable population, the segment of the population most prone to suffer damage during a climate event [42,109]. The results of this sub-index, represented in (Fig 6), indicate high susceptibility in the municipality of Bonanza; medium in Puerto Cabezas, Siuna, Mulukukú; and low in Waspám, Rosita, Prinzapolka, and Waslala.

The susceptibility analysis, in line with the proposed theoretical framework, reaffirms the usefulness of using mixed methodologies and bottom-up approaches that complement statistical information with territorial knowledge, enabling more effective planning for local realities [110]. This perspective is especially relevant in rural and dispersed territories such as the RACCN, where the results show marked differences between municipalities.

Bonanza, for example, presents the highest level of susceptibility due to the combined high proportion of pregnant women and people with disabilities, who together constitute approximately one-third of its total population. Although the municipality has a significant number of local brigades, their effectiveness must be assessed in terms of their actual coverage and response capacity, which may be limited by territorial dispersion.

Municipalities with medium susceptibility, such as Puerto Cabezas, Siuna, and Mulukukú, show a relative balance among the considered indicators. In the case of Mulukukú, the low proportion of the vulnerable population is offset by the small number of local brigades, which partially raises its susceptibility level. Puerto Cabezas and Siuna, on the other hand, combine higher proportions of vulnerable population with high population density, justifying their classification in this category despite the availability of local brigades.

In contrast, municipalities with low susceptibility present more favorable conditions: Rosita has a considerable number of local brigades, while its proportion of vulnerable population remains low. Waslala and Waspám stand out for their low proportion of vulnerable population and the presence of a relatively high number of local brigades, constituting a key protective factor to reduce their level of susceptibility. Prinzapolka, for its part, has the lowest levels of vulnerable population, justifying its classification in this category. However, it is necessary to consider that both Prinzapolka and Waspám have large territorial areas (7,020 km² and 9,341 km², respectively) and very low population density (7 and 11 inhabitants/km²), which implies a high dispersion of settlements. This may limit the coverage and effectiveness of local brigades in emergencies, especially in attending to vulnerable people.

The results obtained for this sub-index corroborate that vulnerability in the RACCN is highly determined by social and territorial factors of a structural nature, which is consistent with the specialized literature on risk studies. Several authors have argued that vulnerability to extreme events does not depend solely on the physical hazard but is shaped by pre-existing inequalities [34,51] and the geographic location of communities [40,74]. These structural conditions explain why municipalities such as Bonanza, Puerto Cabezas, Siuna, and Mulukukú present higher levels of susceptibility to TCs. As Cutter points out, the vulnerability of a place is intrinsically linked to the characteristics of its population and environment; therefore, dispersed territorial units with a high proportion of vulnerable population and limited services, such as the aforementioned municipalities are more prone to disasters [74].

#### 4.2.3 Adaptive capacity sub-index.

The adaptive capacity sub-index was calculated using indicators related to the structural and institutional capacity of municipalities to respond to and recover from the impact of TCs. The indicators grouped under this factor are the density of the road network and the available social infrastructure, represented by the number of school units per thousand inhabitants.

**Fig 7:**
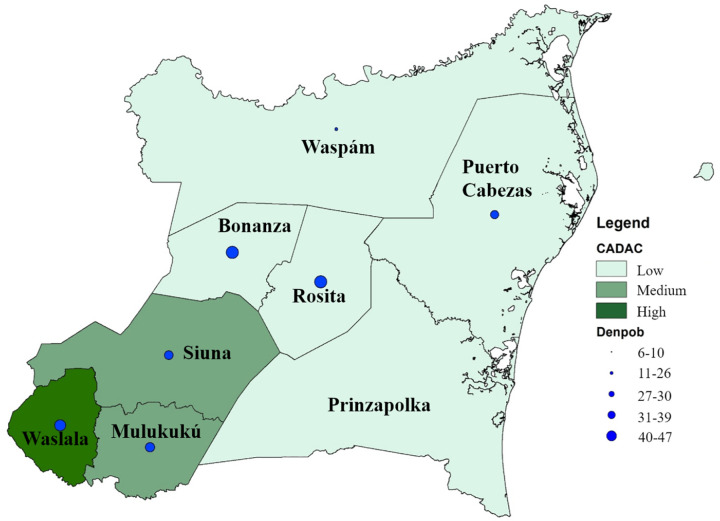
Map of adaptative capacity sub-index. Geographical distribution of the adaptive capacity sub-index in the municipalities of the RACCN. Higher values indicate greater potential to cope with and recover from the impacts of tropical cyclones.

According to the results shown in (Fig 7), the municipality with the highest adaptive capacity is Waslala, favored by a high density of passable roads and a more distributed educational network, which facilitates access to essential services and reduces isolation in emergencies. Mulukukú and Siuna are at the medium level, due to the balance between their indicators. Siuna shows outstanding educational coverage and acceptable road density, and Mulukukú, although with more balanced values, also exceeds the regional average in both aspects.

In contrast, municipalities with low adaptive capacity show unfavorable combinations in one or both indicators. Bonanza and Waspám have low values for both road density and educational infrastructure. Although it has a more developed road network, Rosita has a weak educational infrastructure for its total population. Puerto Cabezas, with a considerable road density but with few school units per thousand inhabitants, significantly limits its institutional capacity. Prinzapolka records the lowest road density in the RACCN, reinforced by its physical isolation from the other municipalities, despite having a relatively high value in educational infrastructure, probably concentrated in urban centers or specific population clusters.

These results highlight significant territorial disparities in adaptive capacity, especially in municipalities such as Rosita, Bonanza, and Prinzapolka, where the combination of low road density and limited educational infrastructure undermines the response to TCs. This limited capacity is particularly critical in dispersed rural areas, where geographic conditions aggravate isolation and hinder recovery. In line with Füssel and Klein, these limitations confirm that adaptive capacity is directly influenced by factors such as institutional strengthening, adequate legal frameworks, information networks, community awareness, and access to essential services [41]. Therefore, this dimension is closely linked to development planning processes, the promotion of social equity, and the strengthening of territorial governance.

Fischer and Frazier argue that the adaptive capacity sub-index in climate change vulnerability assessments allows identifying those municipalities that need greater strategic attention in the future, reinforcing the need for differentiated institutional and policy interventions [87]. In this regard, the municipalities with the lowest adaptive capacity – Bonanza, Waspám, Puerto Cabezas, Rosita, and Prinzapolka – should be prioritized in such interventions. From an operational perspective, Gallopín suggests that improving the adaptive capacity of a system also enables the maintenance or enhancement of quality of life for its members in a given environment, which involves both access to material resources and the consolidation of institutional processes [67]. Therefore, strengthening adaptive capacity is essential to advance towards models of resilience in highly exposed and socially fragile contexts such as the RACCN.

In summary, the analysis of the three sub-indices (exposure, susceptibility, and adaptive capacity) shows the territorial heterogeneity of the RACCN’s vulnerability to TCs, as well as the structural factors that shape it. Each component provides an essential and complementary dimension of the analyzed phenomenon. Exposure reflects the natural hazard conditions (the TCs), susceptibility highlights social and demographic fragilities, and adaptive capacity exposes the institutional strengths and weaknesses that affect the local response. This analysis forms the basis for the construction of the TCs vulnerability index (VItc), which synthesizes these dimensions into a single measure that facilitates comparison among municipalities and the identification of priorities for action.

#### 4.2.4 Tropical Cyclone Vulnerability Index (VItc).

**Fig 8:**
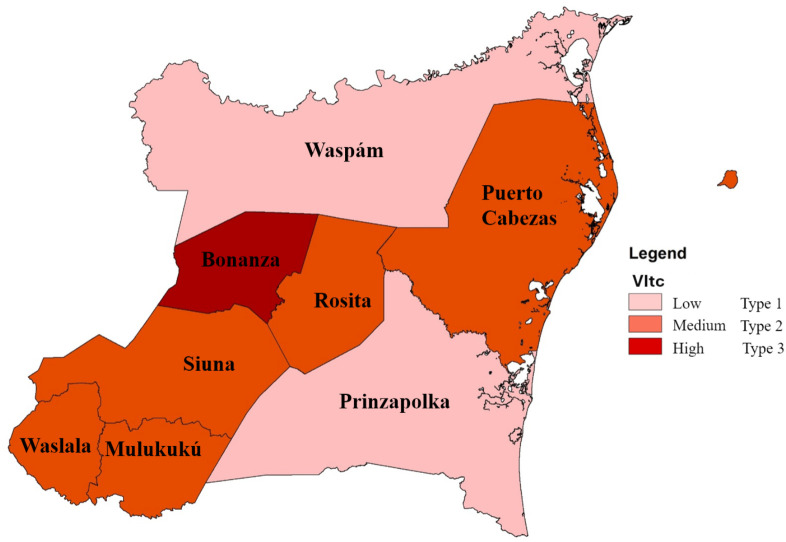
Map of the Vulnerability Index to Tropical Cyclones (VItc) and municipal typology in the RACCN. This map illustrates the geographic distribution of vulnerability alongside the municipal typology, facilitating the identification of the most critical municipalities. The figure enables the identification of priority areas for risk reduction and resource allocation, categorizing municipalities into groups according to the values of VItc.

The VItc was calculated by integrating the normalized sub-indices of exposure, susceptibility, and adaptive capacity, which show differentiated patterns of vulnerability among the municipalities of the RACCN. Quantitatively, Table 2 presents the disaggregated values of each sub-index by municipality, together with the aggregate value of the VItc and its corresponding classification as low, medium, or high. Spatially, the distribution of vulnerability and the resulting municipal typology are shown in (Fig. 8).

**Table 2:** Normalized values of exposure, susceptibility, adaptive capacity, vulnerability index, and classification level sub-indices by municipality in the RACCN.

Municipalities	Ex	Susceptibility	Adaptive Capacity	IVtc	Vulnerability level
**Bonanza**	1.000	1.000	0.038	1.000	High
**Mulukukú**	0.712	0.167	0.444	0.297	Medium
**Prinzapolka**	0.000	0.110	0.234	0.011	Low
**Puerto Cabezas**	0.352	0.213	0.003	0.273	Medium
**Rosita**	0.956	0.081	0.117	0.424	Medium
**Siuna**	0.436	0.228	0.592	0.182	Medium
**Waslala**	0.988	0.090	1.000	0.241	Medium
**Waspám**	0.001	0.000	0.000	0.000	Low

Source: Own elaboration.

The results obtained show that the municipality with the highest level of vulnerability is Bonanza, with maximum values in both the exposure (1.0) and susceptibility (1.0) sub-indices, and a very low adaptive capacity (0.038). This combination of high exposure and social fragility, together with limitations in physical and social infrastructure, positions it as the most critical municipality in the RACCN.

Municipalities with medium vulnerability include Waslala, Mulukukú, Siuna, Rosita, and Puerto Cabezas, while Waspám and Prinzapolka exhibit low vulnerability. This group of municipalities shows differentiated patterns. For instance, Rosita, despite having low susceptibility (0.081), has high exposure (0.956) and low adaptive capacity (0.117), which places its VItc at 0.424. Puerto Cabezas has almost no adaptive capacity (0.003), which, despite its medium exposure (08.352) and moderate susceptibility, raises its overall vulnerability. In contrast, Siuna and Mulukukú have relatively high adaptive capacity (0.444 and 0.592, respectively), which partially offsets their exposure and susceptibility levels. Waslala, on the other hand, combines very high exposure (0.988) with low levels of susceptibility (0.09) but high adaptive capacity (1.0), which moderates its overall vulnerability.

Prinzapolka and Waspám have the lowest VItc values. In the case of Prinzapolka, its low levels of exposure and susceptibility, together with a moderate adaptive capacity, explain this classification. However, it is important to consider that it is a municipality with a large territorial extension, low population density, and high rurality, which could limit its emergency response capacity in practice, despite the favorable values in the model.

Waspám, which has the lowest VItc values, its interpretation requires a more in-depth analysis and particularly careful consideration. This result is mainly explained by the extremely low normalized values across all three sub-indices considered in the model: exposure (0.001), susceptibility (0.000), and adaptive capacity (0.000). In particular, low population density and limited infrastructure availability significantly contribute to the reduction of the composite index value.

However, this result should not be interpreted as an absence of structural vulnerability. While these values reflect the available data used in the model, they must be contextualized in light of the territorial characteristics of the municipality. Waspám is Nicaragua’s largest municipality by land area and is characterized by high rural dispersion, geographic isolation, and limited accessibility to numerous communities. These territorial conditions can increase the difficulties of institutional response and access to services during extreme events, even when the indicators included in the index display low values. In this regard, the apparently low vulnerability value observed in Waspám should be interpreted with caution, as it may only partially reflect the limitations of the available indicators in capturing key territorial dimensions such as spatial isolation, population dispersion, and mobility restrictions. Therefore, complementing the quantitative analysis with perspectives that incorporate territorial, social, and spatial dynamics is essential to better understand the municipality’s actual vulnerability.

In general terms, the VItc results demonstrate that vulnerability does not depend exclusively on a single component, but rather on the balance between exposure to hazards, internal conditions of susceptibility, and the effective capacity to respond and adapt. Although some authors have warned that vulnerability is not easily measurable and that it should be understood as a complex and multifaceted system, emerging from the interaction between social and environmental systems [40,46,71], the value of the VItc, in this study, lies precisely in its capacity to synthesize diverse information and comparatively represent the territorial complexity of the 8 municipalities of the RACCN. This usefulness has also been evidenced in previous studies conducted in other regions exposed to adverse climate change events, particularly TCs [7,12,13,79,80,111].

#### 4.2.5 Sensitivity analysis of the vulnerability index.

To assess the robustness of the tropical cyclone vulnerability index (VItc), a sensitivity analysis was conducted by comparing the values obtained through exploratory factor analysis (EFA) with alternative estimates derived from a principal component analysis (PCA) using jackknife and bootstrap resampling procedures.

**Fig 9:**
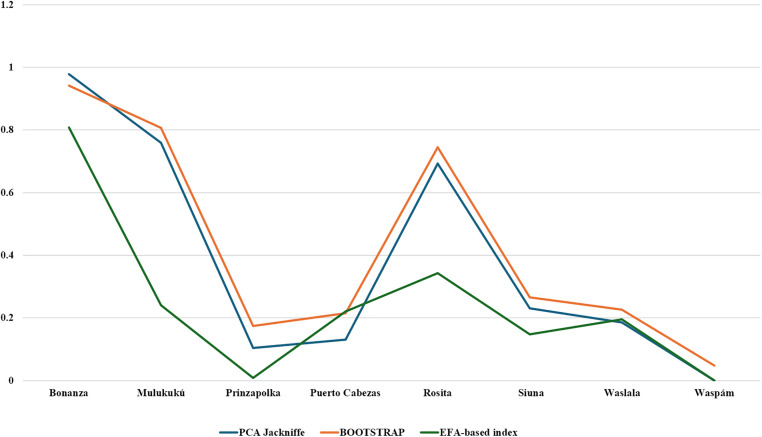
Sensitivity analysis of vulnerability index across estimation methods. This Figure presents a comparison between the index values obtained using EFA and those derived from PCA with jackknife and bootstrap procedures, demonstrating high consistency among the estimates.

The results shown in (Fig 9) and S3 Table show consistent patterns among the values obtained using the different estimation methods. In particular, the municipalities with the highest vulnerability levels in the index derived from EFA also maintain similar positions in the PCA-based estimates with resampling. For example, Bonanza consistently exhibits the highest vulnerability levels across all three estimation methods, while Waspám and Prinzapolka consistently record the lowest index values. Likewise, municipalities such as Rosita, Siuna, and Waslala maintain a very similar trend across the three models, indicating a relatively stable position within the identified vulnerability structure.

Minor variations are observed in Puerto Cabezas, where the EFA-based index presents a slightly higher value compared to the estimates obtained via PCA, while Mulukukú shows a relative decrease in the value estimated via EFA. However, these differences do not alter the general spatial pattern of vulnerability among the municipalities, suggesting that the structure of the VItc is relatively stable in the face of variations in estimation methods and does not depend critically on the weighting scheme used.

In addition, the sensitivity of the cartographic classification of the vulnerability index was evaluated with respect to different methods of grouping the VItc values. The comparison was made between three cartographic classification methods: Natural Breaks (Jenks), Quantiles, and Standard Deviation (see S3 Appendix). Table 3 shows the resulting municipal classification under each grouping scheme.

**Table 3:** Classification of VItc according to different classification methods.

Municipalities	VItc_norm	Jenks	Quantiles	SD
**Bonanza**	1.000000	High	High	High
**Mulukukú**	0.296633	Medium	Medium	Medium
**Prinzapolka**	0.010757	Low	Low	Low
**Puerto Cabezas**	0.272626	Medium	Medium	Medium
**Rosita**	0.424077	Medium	High	Medium
**Siuna**	0.182060	Medium	Low	Medium
**Waslala**	0.241145	Medium	Medium	Medium
**Waspám**	0.000000	Low	Low	Low

Source: Own elaboration.

The results indicate that, although the class break values vary across methods, the overall structure of the municipal vulnerability pattern remains largely consistent. Bonanza consistently appears in the high vulnerability category across all three classification methods, while Prinzapolka and Waspám remain in the low vulnerability category. Likewise, Mulukukú, Puerto Cabezas, and Waslala are placed in the medium vulnerability category regardless of the classification method used.

The only variations between methods are observed in Rosita and Siuna, reflecting the inherent differences between the cartographic classification algorithms. These differences are mainly explained by the nature of the quantile method, which distributes observations uniformly across classes without necessarily considering the natural structure of the data [112,113]. Taken together, these results indicate that the spatial pattern of the index is stable across different classification schemes.

#### 4.2.6 Typology of the municipalities of the RACCN.

Building on the vulnerability levels obtained in the VItc, a typology of municipalities was developed to reflect differentiated patterns of vulnerability to TCs in the RACCN. According to Sánchez-Zamora et al. [60] the functionality of a municipal typology lies in its ability to capture existing territorial particularities, grouping municipalities with similar [vulnerability] patterns, thereby facilitating the design of tailored intervention strategies.

The typology distinguishes three types of municipalities (Fig 8), each representing a distinct combination of exposure, susceptibility, and adaptive capacity:

*Type 1 municipalities,* represented by Bonanza, are characterized by high exposure to TCs, high population density, high climatic and demographic pressure, and limited institutional and social response infrastructure. They present the highest values of the vulnerability index and require urgent priority and targeted intervention to strengthen risk management and enhance local and institutional capacities.

*Type 2 municipalities*, made up of Waslala, Mulukukú, Siuna, Rosita, and Puerto Cabezas, represent territories with intermediate vulnerability. These territories combine moderate to high exposure with a differentiated presence of vulnerable population, and relatively stronger structural and institutional conditions, such as road networks and educational infrastructure. They need integrated strategies that consolidate advances in response, reinforce resilience, and adaptive capacity, while reducing internal disparities.

*Type 3 municipalities,* comprising Waspám and Prinzapolka, are defined by low exposure, large territorial extensions, low population density, and predominantly rural settings, which leads to the assertion that there is an elevated level of geographic dispersion. They have low levels of vulnerability according to the VItc, although they face structural challenges not fully captured by the VItc, such as geographic isolation and reliance on river transport. These municipalities require interventions adapted to the territorial context, prioritizing connectivity, equity in access to services, and institutional strengthening adapted to their territorial realities.

This typology enables a more nuanced understanding of vulnerability across the RACCN, moving beyond aggregate index values to inform differentiated and context-sensitive policy responses. This classification allows not only to facilitate the establishment of a vulnerability hierarchy, but also the geographic prioritization of actions, in line with previous studies [87] that emphasize the importance of reducing exposure and susceptibility while enhancing adaptive capacity in the face of future climate impacts.

## 5. Conclusions

This study assessed the vulnerability of the RACCN to TCs through the development of a composite index (VItc) based on the dimensions of exposure, susceptibility, and adaptive capacity. The main objective was to identify the most vulnerable municipalities and provide an analytical tool to guide risk management strategies related to TCs. The frequency and intensity of these events in the RACCN, as observed over the past three decades, underscores the urgent need to strengthen prevention and response mechanisms, as well as to evaluate the effectiveness of existing policies and regulatory frameworks in the most exposed municipalities.

The analysis of the exposure, susceptibility, and adaptive capacity sub-indices, in conjunction with municipal typology, provides a useful analytical framework to guide the design and implementation of targeted intervention strategies. The results highlight significant territorial disparities among the municipalities of the RACCN and reveal structural inequalities in the spatial distribution of risk. In this regard, the findings contribute to identifying priority areas for intervention and could support the formulation of risk management policies that are more sensitive to territorial equity, the prioritization of emergency response areas, and the strengthening of community resilience.

These results also demonstrate the potential of composite indices as a diagnostic tool for identifying priority areas in the RACCN, highlighting notable disparities between municipalities such as Waslala, characterized by relatively higher adaptive capacity, and Prinzapolka, characterized by high levels of physical isolation. As noted by Eakin and Luers [71] and Emrich and Cutter [42], these indices are especially valuable for understanding vulnerability patterns in rural territories where climate-sensitive livelihoods and environmental conditions increase sensitivity to climate change. However, these disparities should be interpreted with caution, given that some factors relevant to understanding social and territorial inequalities may not have been fully captured by the available indicators.

The methodology used in this study, based on a literature review, factor analysis, and geospatial mapping, could be adapted to other regions with similar characteristics, provided that comparable public data is available. In this regard, the spatial representation of the VItc used to visualize territorial vulnerability patterns among municipalities in the RACCN could help guide disaster risk planning and management processes at the regional level. The maps derived from the VItc facilitate the identification of territories with higher relative levels of vulnerability, which may support the prioritization of interventions related to prevention, preparedness, and adaptation to the impact of TCs. However, the effective use of these results as a decision-making support tool requires verification and further validation through dialogue with institutional actors, policymakers, and local communities.

Vulnerability assessments require a high level of analysis of spatial data and territorial indicators, which represents a methodological challenge when information is limited. In this context, the assessment process presented several limitations that should be considered when interpreting the results. First, the availability of statistical information at more disaggregated territorial scales constitutes one of the main constraints of the analysis. Although vulnerability to TCs can manifest differently at the neighborhood or community level, the smallest scale of territorial organization, the available data for the RACCN were organized exclusively at the municipal level at the time this research was conducted. Consequently, the vulnerability index was constructed using the eight municipalities in the region, which represent the entirety of the available territorial universe. Changes in the territorial scale or the incorporation of new variables could modify the relative levels of vulnerability identified.

Second, the selection of indicators was constrained by the availability of comparable data across municipalities. Although the variables included are supported by the theoretical framework and have been widely used in vulnerability studies, it was not possible to validate them in context through participatory processes with local stakeholders during the indicator selection phase of this study. Likewise, some potentially relevant indicators could not be incorporated due to the limited availability of standardized statistical information for the region. These include variables related to the health sector, such as the spatial distribution of healthcare facilities or epidemiological conditions. Although the indicator of health facilities per thousand inhabitants was initially considered as a measure of adaptive capacity, it was not retained in the final index due to its low statistical contribution in the EFA. Similarly, indicators linked to local livelihoods could not be included either, particularly those related to dependence on climate-sensitive economic activities, such as agriculture and livestock farming, which can significantly influence the susceptibility of territories to TCs.

Third, some variables used in the index were defined based on approximations available at the municipal level. For example, population density was used as a proxy for demographic exposure, as it reflects the concentration of people potentially affected by TCs. However, this indicator does not fully capture physical exposure at the micro-territorial level, which is determined by factors such as proximity to flood-prone areas or coastal areas. Consequently, exposure may be underestimated in sparsely populated rural areas that are highly exposed to natural hazards.

A fourth limitation relates to the difficulty of adequately capturing territorial connectivity, rural dispersion, and spatial isolation through the available indicators. In municipalities with very low population density and large territorial areas, such as Waspám and Prinzapolka, whose VItc results classify them as having relatively low levels of vulnerability, this condition may be partially underestimated. This is because factors such as the distance between settlements and effective access to transportation or communication infrastructure are not fully represented in the indicators included in the index. Consequently, although the VItc allows the identification of relative vulnerability patterns among municipalities, the interpretation of these results must consider the particular territorial characteristics of these dispersed rural contexts [42,71].

Furthermore, as with other composite vulnerability indices, the results depend on the variables selected, the aggregation methods applied, and the statistical procedures used to estimate the weights of the indicators. Although sensitivity analyses were conducted in this study to assess the stability of the index, different methodological decisions could lead to variations in the obtained values. Therefore, the index should be interpreted as an analytical approximation of territorial vulnerability based on available information, rather than as a comprehensive representation of all the dimensions shaping vulnerability to TCs.

This study also opens up new lines of research, including the assessment of economic impacts on infrastructure and livelihoods; the analysis of forced displacement and social conflicts; and studies focused on mental health, community dynamics, and humanitarian assistance. Future research could incorporate additional indicators and participatory approaches to strengthen contextual validation and more accurately capture the factors that shape territorial vulnerability. Similarly, the inclusion of indicators related to territorial accessibility and socioeconomic conditions, including variables related to income, poverty, or educational attainment, as well as gender-related factors and health conditions would improve the representation of the multiple dimensions that shape vulnerability in dispersed rural contexts.

It is also important to explore integrative approaches within development planning that incorporate environmental and social variables and adopt participatory methods reflecting local knowledge and lived experiences. In particular, further research should examine sustainable natural resource management strategies to reduce structural vulnerability, and to evaluate the effectiveness and appropriation of early warning systems, considering technological, institutional, and socio-cultural barriers. Future research could also incorporate additional gender-related indicators and participatory approaches to better capture the differentiated vulnerabilities experienced by women and other socially vulnerable groups during climate extremes, proposing gender-sensitive approaches to adaptation and recovery. Finally, understanding local governance mechanisms, institutional capacity, and citizen participation is essential for strengthening risk management and moving toward territories that are more resilient to the effects of TCs.

Taken together, the findings of this study provide empirical evidence of the spatial patterns of vulnerability to TCs in the RACCN and generate relevant information for stakeholders in the public and private sectors, as well as for researchers and academics interested in disaster risk reduction. In this way, the study expands the empirical evidence on territorial climate vulnerability assessment in rural regions highly exposed to TCs, offering a replicable methodological approach for contexts with data limitations.

## Supporting information

S1 Table Tropical cyclones that directly or indirectly affected Nicaragua between 1988 and 2022. This table includes the cyclone name, its classification according to the Saffir–Simpson Hurricane Wind Scale, indicating the intensity of the event (e.g., tropical wave (TW), tropical depression (TD), tropical storm (TS), and hurricane categories H1–H5), as well as the month and year of occurrence, and the general region of impact (landfall) for each event that affected Nicaragua during the study period. Data was compiled from EM-DAT and NOAA’s Hurricane Research Division.
(PDF)

S2 Table Normalized values of the indicators used to construct the exposure, susceptibility, and adaptive capacity sub-indices for each municipality in the RACCN. This table presents the normalized values (0–1 scale) of the indicators used in the construction of the vulnerability sub-indices (exposure, susceptibility, and adaptive capacity) for each municipality in the North Caribbean Coast Autonomous Region of Nicaragua (RACCN). These normalized values constitute the input data used to calculate the respective sub-indices and the final Tropical Cyclone Vulnerability Index (VItc). Each column corresponds to a specific indicator, and each row represents a municipality included in the analysis.
(PDF)

S1 Appendix Procedure for calculating the accumulated precipitation indicator between 1988–2022. This appendix details the methodology used to construct the accumulated precipitation indicator for the period 1988–2022.
(PDF)

S2 Appendix Cumulative precipitation at RACCN for the period 1988–2022. During the 1988–2022 period, more than 18,900 mm were recorded from 30 cyclonic events. The analysis includes normalization by municipal surface area to ensure spatial comparability.
(PDF)

S3 Table Sensitivity analysis across estimation methods. This table presents the values of the Tropical Cyclone Vulnerability Index (VItc) estimated using three different methodological approaches: an Exploratory Factor Analysis (EFA)-based weighting scheme, Principal Component Analysis (PCA) combined with jackknife resampling, and bootstrap resampling. These values correspond to the data used to construct Fig 9 and allow for a direct comparison of the index results across alternative estimation methods.
(PDF)

S3 Appendix Sensitivity of the cartographic classification of the vulnerability index. S3 Appendix presents a sensitivity analysis of the cartographic classification of the Tropical Cyclone Vulnerability Index (VItc). It includes the class break values derived from three different classification methods: Natural Breaks (Jenks), quantiles, and standard deviation, as well as the resulting classification of municipalities under each approach.
(PDF)
