# Supplementary material for: Vulnerability assessment to tropical cyclones in the North Caribbean Coast of Nicaragua (1988–2022)
Source: PLoS One. 2026 Jun 22;21(6):e0352206. doi: 10.1371/journal.pone.0352206 (PMC13286158; doi:10.1371/journal.pone.0352206)
Supplement: S1 Table — This table includes the cyclone name, its classification according to the Saffir–Simpson Hurricane Wind Scale, indicating the intensity of the event (e.g., tropical wave (TW), tropical depression (TD), tropical storm (TS), and hurricane categories H1–H5), as well as the month and year of occurrence, and the general region of impact (landfall) for each event that affected Nicaragua during the study period. Data was compiled from EM-DAT and NOAA’s Hurricane Research Division. (PDF) [file pone.0352206.s001.pdf]

S1 Table. Tropical cyclones that directly or indirectly affected Nicaragua between 1988 and 2022

No.	SS Scale	Name	Year	Month	Landfall
1	H4	Joan	1988	October	RACCS
2	TS	Bret	1993	August	RACCS
3	TS	Gert	1993	September	RACCS
4	TD	Gordon	1994	November	CARIBBEAN
5	TD	Roxanne	1995	October	CARIBBEAN
6	H1	Cesar	1996	July	RACCS
7	H5	Mitch	1998	October	Honduras
8	TS	Katrina	1999	October	CARIBBEAN
9	TS	Keith	2000	September	CARIBBEAN
10	TD	No. 9	2001	September	CARIBBEAN
11	TD	Michelle	2001	October	RACCN
12	TS	Isidore	2002	September	CARIBBEAN
13	H3	Beta	2005	May	RACCS
14	TS	Stan	2005	October	CARIBBEAN
15	TD	Adrian	2005	May	PACIFICO
16	H5	Felix	2007	September	RACCN
17	TS	Alma	2008	May	PACIFICO
18	H1	Ida	2009	November	RACCN
19	TW	Alex	2010	June	CARIBBEAN
20	TS	Matthew	2010	September	RACCN
21	TS	Paula	2010	October	RACCN
22	TW	Helene	2012	August	RACCN
23	TW	Barry	2013	June	RACCN
24	TS	Hanna	2014	October	RACCN
25	H3	Otto	2016	November	RACCS
26	TS	Nate	2017	October	RACCN
27	H4	Eta	2020	November	RACCN
28	H4	Iota	2020	November	RACCN
29	TS	Bonnie	2022	July	RACCS
30	H1	Julia	2022	October	RACCS
