# Supplementary material for: Vulnerability assessment to tropical cyclones in the North Caribbean Coast of Nicaragua (1988–2022)
Source: PLoS One. 2026 Jun 22;21(6):e0352206. doi: 10.1371/journal.pone.0352206 (PMC13286158; doi:10.1371/journal.pone.0352206)
Supplement: S2 Table — This table presents the normalized values (0–1 scale) of the indicators used in the construction of the vulnerability sub-indices (exposure, susceptibility, and adaptive capacity) for each municipality in the North Caribbean Coast Autonomous Region of Nicaragua (RACCN). These normalized values constitute the input data used to calculate the respective sub-indices and the final Tropical Cyclone Vulnerability Index (VItc). Each column corresponds to a specific indicator, and each row represents a municipality included in the analysis. (PDF) [file pone.0352206.s002.pdf]

S2 Table. Normalized values of the indicators used to construct the exposure, susceptibility, and adaptive capacity sub-indices for each municipality in the RACCN.

Municipality	EXP1PA	EXP2PC	EXP3DP	SC1PR	SC2PG	SC3CH	SC4EL	SC5DIS	CA1RV	CA2US	CA3SC	CA4BL
**Bonanza**	0.893	0.241	0.975	0.229	1.000	0.532	0.338	1.000	0.185	0.026	0.115	1.000
**Mulukukú**	0.716	1.000	0.638	0.771	0.021	0.025	0.000	0.000	0.565	0.111	0.472	0.051
**Prinzapolka**	0.099	0.162	0.000	1.000	0.000	0.135	0.617	0.025	0.000	0.296	0.457	0.133
**Puerto Cabezas**	0.233	0.000	0.500	0.000	0.015	0.252	1.000	0.018	0.334	0.352	0.000	0.000
**Rosita**	0.795	0.065	1.000	0.521	0.007	0.719	0.261	0.065	0.424	0.000	0.108	0.222
**Siuna**	0.305	0.060	0.576	0.750	0.064	1.000	0.108	0.023	0.363	0.189	0.758	0.037
**Waslala**	1.000	0.548	0.837	0.125	0.014	0.016	0.506	0.048	1.000	1.000	1.000	0.200
**Waspám**	0.000	0.122	0.108	1.000	0.012	0.000	0.473	0.012	0.201	0.659	0.057	0.283
