# Supplementary material for: Vulnerability assessment to tropical cyclones in the North Caribbean Coast of Nicaragua (1988–2022)
Source: PLoS One. 2026 Jun 22;21(6):e0352206. doi: 10.1371/journal.pone.0352206 (PMC13286158; doi:10.1371/journal.pone.0352206)
Supplement: S1 Appendix — This appendix details the methodology used to construct the accumulated precipitation indicator for the period 1988–2022. (PDF) [file pone.0352206.s003.pdf]

S3 Appendix. Procedure for calculating the accumulated precipitation indicator between 1988-2022.

For the calculation of the accumulated precipitation indicator, the following procedure was performed: identification of the cyclonic events from the EMDAT data download; classification of the events according to their intensity into tropical disturbances, depressions, storms and hurricanes; determination of the access point to the Nicaraguan territory by contrasting this information with the official reports from the NHC archive; determination of the number of days of influence[1] for each event, taking as midpoint the date reported in the official reports (Hurricane Data by Year (noaa.gov)) (NOAA, 2018); and downloading the images from the CHIRPS dataset for the period corresponding to each tropical cyclone obtaining 30 periods, equivalent to the number of events analyzed. For image processing, the GEE platform (Gorelick et al., 2017) is used (Zhao et al., 2021) by applying a filter command. filter(ee.Filter.date) to the CHIRPS 2 database. From the above, 30 synthetic images corresponding to each cyclone reported were obtained. These were summed to obtain the accumulated rainfall of the evaluated events. Subsequently, a grouping by ranges of equal distance was performed to zone the recurrence in 5 classes ranging from very low to very high according to the rainfall accumulation. To calculate the daily image of meteorological events between 1988 and 2022) the following equation was applied:

$$R_{Ac}(p) = \sum_{i=1}^{n} R_i(p)$$

Where;

1. $R_{(Ac)}$ (p) is the resulting synthetic image of accumulated precipitation for the *period of* the event.

[1] This information varies according to each event since the passage of some tropical cyclones over the study area did not have the same duration in terms of days.

2. $R_i(p)$ is the daily image for the *event* period corresponding to the day i
3. The summation from $i = 1$ to $i = n$ indicates that the daily images are being summed to obtain the total synthetic image for the cyclone event.

For the total accumulated rainfall during the 30 years evaluated (RACCN accumulated precipitation from 1988 to 2022), the following equation was used:

$$R_{Tot}(p) = \sum_{j=0}^{T} \left(\sum_{i=1}^{n} R_i(p,j) \right)$$

Where;

1. $R_{(Tot)}(p)$ is the resulting synthetic image for the period 1988 to 2022.
2. $R_i(p,j)$ is the daily image for event j of period p corresponding to day i.
3. The first summation $\sum_{i=1}^{n}$ is performed on the daily images for each event j.
4. The second summation $\sum_{j=0}^{T}$ is performed for the consolidation of all synthetic images for each period over the 30 events (T).
