# Supplementary material for: Vulnerability assessment to tropical cyclones in the North Caribbean Coast of Nicaragua (1988–2022)
Source: PLoS One. 2026 Jun 22;21(6):e0352206. doi: 10.1371/journal.pone.0352206 (PMC13286158; doi:10.1371/journal.pone.0352206)
Supplement: S2 Appendix — During the 1988–2022 period, more than 18,900 mm were recorded from 30 cyclonic events. The analysis includes normalization by municipal surface area to ensure spatial comparability. (PDF) [file pone.0352206.s004.pdf]

S4 Appendix. Cumulative precipitation at RACCN for the period 1988-2022.

The analysis and processing of the CHIRPS (Climate Hazards Group InfraRed Precipitation with Station data) dataset made it possible to calculate the cumulative precipitation associated with the 30 cyclonic events recorded between 1988 and 2022. In total, an accumulation of more than 18,900 mm was estimated during the period analyzed. By decades, the accumulated values were as follows: 1990-1999: 7 events, 5,617.77 mm; 2000-2009: 10 events, 5,471.26 mm; 2010-2019: 8 events, 3,937.44 mm, 2020-2022: 4 events, 3,880.16 mm.

The total precipitation was normalized according to the surface area of each municipality, to avoid biases derived from the size of the territory. In this way, exposure is not only evaluated in terms of the total volume received, but also in relation to its spatial distribution and population density, which allows a more accurate estimation of the potential risk to tropical cyclones at the municipal scale.

The event with the highest individual precipitation was Hurricane Joan (1988), with 999.62 mm. The year with the highest rainfall was 2020, with 2,878.31 mm, mainly associated with hurricanes Eta and Iota (category 4). In contrast, the lowest record was in 2002, with 279.27 mm caused by tropical storm Isidore, which, although it maintained its trajectory over the waters of the Caribbean Sea, caused 2 deaths, about 300 people affected and more than one million dollars in damages. During the 32 years analyzed, the municipalities that recorded the highest accumulated volumes of precipitation were Puerto Cabezas, Waspám, Prinzapolka, Rosita and Bonanza, the latter two in relatively smaller proportions.

Graph 1: Accumulated precipitation by decades and municipality.

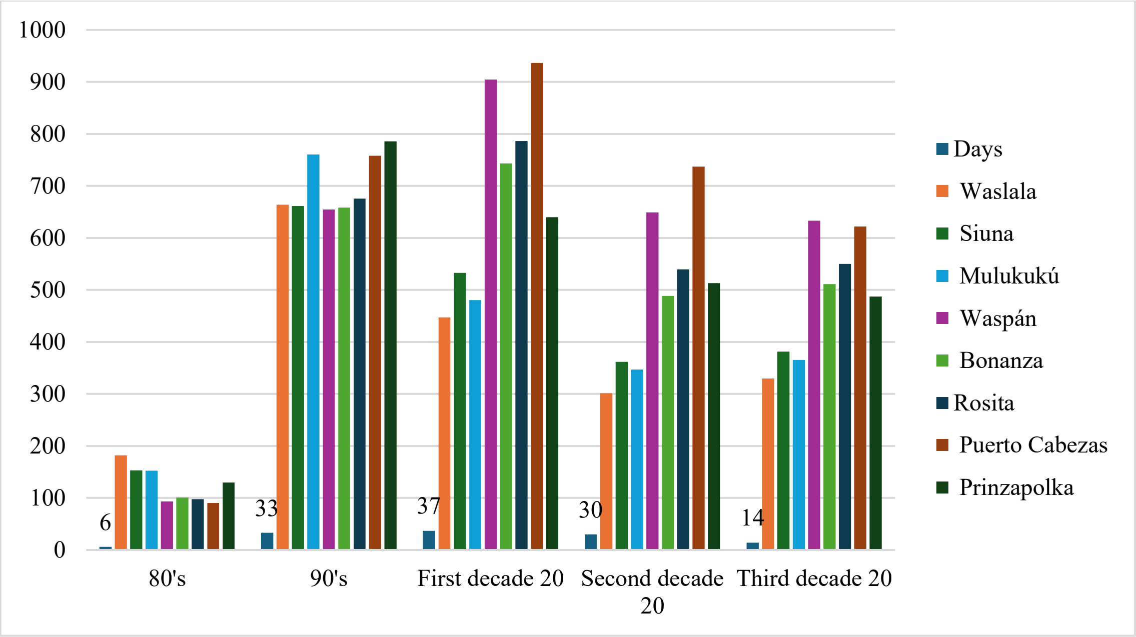

Source: Own elaboration
