# Supplementary material for: Vulnerability assessment to tropical cyclones in the North Caribbean Coast of Nicaragua (1988–2022)
Source: PLoS One. 2026 Jun 22;21(6):e0352206. doi: 10.1371/journal.pone.0352206 (PMC13286158; doi:10.1371/journal.pone.0352206)
Supplement: S3 Table — This table presents the values of the Tropical Cyclone Vulnerability Index (VItc) estimated using three different methodological approaches: an Exploratory Factor Analysis (EFA)-based weighting scheme, Principal Component Analysis (PCA) combined with jackknife resampling, and bootstrap resampling. These values correspond to the data used to construct Fig 9 and allow for a direct comparison of the index results across alternative estimation methods. (PDF) [file pone.0352206.s005.pdf]

S5 Table. Sensitivity analysis across estimation methods

Municipalities	Jackknife_PCA	BOOTSTRAP	EFA
Bonanza	0.97847674	0.941396938	0.808610697
Mulukukú	0.758871039	0.807409525	0.240068416
Prinzapolka	0.10357618	0.17404959	0.008989969
Puerto Cabezas	0.130986774	0.214371042	0.220662600
Rosita	0.69237158	0.74529241	0.343082752
Siuna	0.230656517	0.265172535	0.147456691
Waslala	0.185049244	0.226172536	0.195216463
Waspám	0.000610865	0.048105022	0.000294989
