# Supplementary material for: Vulnerability assessment to tropical cyclones in the North Caribbean Coast of Nicaragua (1988–2022)
Source: PLoS One. 2026 Jun 22;21(6):e0352206. doi: 10.1371/journal.pone.0352206 (PMC13286158; doi:10.1371/journal.pone.0352206)
Supplement: S3 Appendix — presents a sensitivity analysis of the cartographic classification of the Tropical Cyclone Vulnerability Index (VItc). It includes the class break values derived from three different classification methods: Natural Breaks (Jenks), quantiles, and standard deviation, as well as the resulting classification of municipalities under each approach. (PDF) [file pone.0352206.s006.pdf]

S6 Appendix. Sensitivity of the cartographic classification of the vulnerability index

Class cut-off values for the different vulnerability index classification methods (VItc_norm).

Class break values			
	**Jenks**	**Quantiles**	**SD**
High	0.424078 - 1.000000	0.296634 - 1.000000	0.451008 - 1.000000
Medium	0.010758 - 0.424077	0.182061 - 0.296633	0.155818 - 0.451007
Low	0.000000 - 0.010757	0.000000 - 0.182060	0.000000 - 0.155817

Note: The classification thresholds were derived from the output generated in ArcMap 10.4.1

Classification of VItc according to different classification methods

**Municipalities**	**VItc_norm**	**Jenks**	**Quantiles**	**SD**
Bonanza	1.000000	High	High	High
Mulukukú	0.296633	Medium	Medium	Medium
Prinzapolka	0.010757	Low	Low	Low
Puerto Cabezas	0.272626	Medium	Medium	Medium
Rosita	0.424077	Medium	High	Medium
Siuna	0.182060	Medium	Low	Medium
Waslala	0.241145	Medium	Medium	Medium
